# Dental Physiology

**Published:** 1879-08

**Authors:** E. Parsons

**Affiliations:** Savannah, Ga.


### ARTICLE VII.

### *Dental Physiology.*

BY DR. E. PARSONS, OF SAVANNAH, GA.

Read before the National Dental Association, July 8th, 1879.

You are aware that many volumes have been written upon this subject. I have neither the time or ability to do justice to it. It is generally divided into Human, Animal and Vegetable. A thorough knowledge of all these is of great advantage to those who are engaged in any branch of the healing art. You will bear with me, however, if I only submit for your consideration a few thoughts on the constitution and use of the glands of the mouth. But in doing this, in order to show their use, without which the subject is a very dry one you will excuse me if I trench a little on the sciences of Chemistry and Pathology. I present for your consideration the glands of the mouth, first simple, second compound, third mucus, fourth serous.

First.—A simple gland consists of a faliculous or small sack of globular form, with blood vessel and nerve, and possessing an absorbant property, by which means it secretes the fluids proper to its office, and by a pulsatory motion it excretes the fluid it has prepared in its little chemical laboratory, through a very minute duct.

Second.—A compound gland is a number of simple glands excreting their contents into a common duct.

The Paratoid, sub-maxillary and sublingual glands furnish the mouth with a good lubricating substance, known as saliva, as it contains more or less lactic acid; when swallowed is a great aid in the ordinary process of digestion. These glands according to the best authors secrete from fourteen to twenty ounces every twenty four hours.

Third.—The whole mouth and tongue is covered with minute mucus glands, each one of which act singly, but in their aggregate are called the mucus membrane. They resemble in their structure the capillary glands of the skin. Their office is to secrete mucus and if not interrupted in their endeavors, they furnish the mouth with a substance much resembling the white of an egg, and gives to its surface a smooth, and agreeable sensation; it also gives a smooth and easy passage of our food to its point of destination.

Fourth.—The serous glands, usually called the serous membrane, are exceedingly small, but capable of absorbing serum from the blood and excreting the same in a highly purified state, slightly acidulated; the substance is transparent and as limpid as the purest water. In the normal state these ducts can only be seen by the aid of a good microscope. Like the mucus glands they are present in all the surfaces of the oral cavity. Their use is to liquify the excreted mucus and prevent it becoming ropy. When these glands become diseased by secreting too much lactic or other acids, the little ducts become inflamed, the mouth of the duct turns outward in a rose form, and often run together and form what is called a canker sore. This in my opinion is the cause of nursing sore mouths. A preparation of Glycerine and Carbolic Acid, say to one ounce of the former add twenty drops of the latter and applied with the finger or a swab, two or three times a day, will usually effect a cure in three or four days. The same is a very efficient remedy for a common sore throat and is almost a sure cure for tonsilitus, if properly applied.

If all the glands of the mouth could be kept in a normal condition, the Dentists' occupation would be gone, unless the breath should become acidulated in the lungs and passing through the mouth should have a deleterious effect on the teeth.

Although the act of breathing and its effect on the glands of the mouth and teeth may seem to be foreign to our subject, a few words on the subject may assist us to better understand, at least one of the causes of diseased action. In the whole animal kingdom, I do not know any animal except man that sleeps with the mouth open. Both nature and revelation teach us that man was made to breathe through his nostrils. While speaking or singing we all more or less inspire and expire through the mouth, but this occupies but a small portion of our time, and here let it be noted, that most incessant talkers have bad teeth.

The air we breathe is composed chiefly of Oxygen, Nitrogen and Carbon, nearly all the Oxygen is retained, and part of the Nitrogen ; the Carbon is expelled when we perform the act called breathing. Let any person inhale the air through the mouth, and the little ducts of the mouth and tongue will close up and effect a cool and dry sensation, but when the air passes through the nostrils no such sensation is experienced. The blood being rarified and in a measure purified in the lungs by Oxygen, the Carbon unites with these impurities and is expelled, producing what we call an acidulated breath, and if the breath is constantly passed off through the mouth as is the case of one sleeping with the mouth open, the superior front incisors must be of extraordinary good quality not to be affected by it; the inferior incisors are protected by the tongue and do not often suffer. Cases frequently present themselves for treatment where we find the superior front teeth decayed, and the back teeth sound, and then again the reverse is the case. I often make inquiry into the habits of such patients, and generally find that the former sleep with their mouths open, and the latter with them closed. The front teeth I conclude are disintegrated by the acidulated breath and the back teeth by an acidulated secretion of the salivary glands.

Again, sometimes one side of the mouth and teeth appear healthy and the other side diseased. Upon inquiry I am generally informed that the person sleeps lying upon the side affected. So long as the secretions and the breath are in a normal condition, it is not important how we sleep, provided we keep our mouth closed. Until we understand all the causes of disease to which the oral cavity is liable, we shall not be prepared to give such advice as our patients stand most in need of.

It is not our purpose in these remarks that you should keep your mouths shut at this meeting, but on the contrary, that you open them and freely ventilate your ideas on this, and all other subjects that may come before us.

---

